# Evaluating an extended rehabilitation service for stroke patients (EXTRAS): study protocol for a randomised controlled trial

**DOI:** 10.1186/s13063-015-0704-3

**Published:** 2015-05-05

**Authors:** Helen Rodgers, Lisa Shaw, Robin Cant, Avril Drummond, Gary A Ford, Anne Forster, Katie Hills, Denise Howel, Anne-Marie Laverty, Christopher McKevitt, Peter McMeekin, Christopher Price

**Affiliations:** Institute of Neuroscience (Stroke Research Group), Newcastle University, 3-4 Claremont Terrace, Newcastle upon Tyne, NE2 4AE England UK; Stroke Northumbria, Northumbria Healthcare NHS Foundation Trust, North Tyneside General Hospital, Rake Lane, North Shields, Tyne and Wear, NE29 8NH England UK; Stroke Northumbria, Northumbria Healthcare NHS Foundation Trust, Wansbeck General Hospital, Woodhorn Lane, Ashington, Northumberland, NE63 9JJ England UK; Lay investigator. Contact via: Institute of Neuroscience (Stroke Research Group), Newcastle University, 3-4 Claremont Terrace, Newcastle upon Tyne, NE2 4AE England UK; Division of Rehabilitation and Ageing, University of Nottingham, B Floor, Medical School, Queen’s Medical Centre, Nottingham, NG7 2UH England UK; Oxford Academic Health Science Network, John Eccles House, Robert Robinson Avenue, Oxford Science Business Park, Oxford, OX4 4GP England UK; Academic Unit of Elderly Care and Rehabilitation, University of Leeds, Bradford Institute for Health, Bradford Royal Infirmary, Duckworth Lane, Bradford, BD9 6RJ England UK; Institute of Health and Society, Newcastle University, Baddiley-Clark Building, Richardson Road, Newcastle upon Tyne, NE2 4AX England UK; Department of Public Health Sciences, King’s College London, Capital House, 7th Floor, 41 Western Street, London, SE1 3QD England UK; Faculty of Health and Life Sciences, Coach Lane Campus, Northumbria University, Newcastle upon Tyne, NE7 7XA England UK

**Keywords:** Stroke, Rehabilitation, Early supported discharge, Community services, Health economics, Randomised controlled trial

## Abstract

**Background:**

Development of longer term stroke rehabilitation services is limited by lack of evidence of effectiveness for specific interventions and service models. We describe the protocol for a multicentre randomised controlled trial which is evaluating an extended stroke rehabilitation service. The extended service commences when routine ‘organised stroke care’ (stroke unit and early supported discharge (ESD)) ends.

**Methods/design:**

This study is a multicentre randomised controlled trial with health economic and process evaluations. It is set within NHS stroke services which provide ESD. Participants are adults who have experienced a new stroke (and carer if appropriate), discharged from hospital under the care of an ESD team.

The intervention group receives an extended stroke rehabilitation service provided for 18 months following completion of ESD. The extended rehabilitation service involves regular contact with a senior ESD team member who leads and coordinates further rehabilitation. Contact is usually by telephone. The control group receives usual stroke care post-ESD. Usual care may involve referral of patients to a range of rehabilitation services upon completion of ESD in accordance with local clinical practice. Randomisation is via a central independent web-based service.

The primary outcome is extended activities of daily living (Nottingham Extended Activities of Daily Living Scale) at 24 months post-randomisation. Secondary outcomes (at 12 and 24 months post-randomisation) are health status, quality of life, mood and experience of services for patients, and quality of life, experience of services and carer stress for carers. Resource use and adverse events are also collected. Outcomes are undertaken by a blinded assessor.

Implementation and delivery of the extended stroke rehabilitation service will also be described. Semi-structured interviews will be conducted with a subsample of participants and staff to gain insight into perceptions and experiences of rehabilitation services delivered or received.

Allowing for 25% attrition, 510 participants are needed to provide 90% power to detect a difference in mean Nottingham Extended Activities of Daily Living Scale score of 6 with a 5% significance level.

**Discussion:**

The provision of longer term support for stroke survivors is currently limited. The results from this trial will inform future stroke service planning and configuration.

**Trial registration:**

This trial was registered with ISRCTN (identifier: ISRCTN45203373) on 9 August 2012.

## Background

There are approximately 300,000 disabled stroke survivors in the UK [1]. Although one third of patients remain disabled 12 months after acute stroke, the longer term provision of stroke rehabilitation is sparse. Input from a therapist or nurse with specialist expertise in stroke rehabilitation is rare beyond 6 months post-discharge. Stroke patients and their informal carers are frequently disappointed and frustrated that longer term rehabilitation is not more widely available, and the UK Stroke Survivor Needs Survey reported that nearly half of stroke survivors experience unmet needs in the longer term [2].

Stroke units and early supported discharge (ESD) services are effective ways to improve patient outcomes and the quality of care following stroke [3,4]. These services are referred to as ‘organised stroke care’, and their key features are multidisciplinary stroke specialist expertise and coordination of care [5,6]. In contrast, there is no clear evidence of the clinical and cost effectiveness of longer term rehabilitation following stroke [7]. A Cochrane review of therapy-based rehabilitation services for patients living at home more than one year after stroke concluded that it was unclear whether rehabilitation provided after one year can improve recovery [8]. However, therapy-based rehabilitation services for stroke patients at home provided soon after discharge have been shown to be effective [9].

ESD services offer patients the opportunity to continue rehabilitation in their own home following a period of stroke unit care. ESD provided by a specialist multidisciplinary team leads to better clinical outcomes, increased satisfaction with care and reduced NHS costs [3]. ESD is a core component of an evidence-based stroke service, and it is the current ‘gold standard’ for an early community rehabilitation service for stroke patients with ongoing rehabilitation needs who are discharged to their own homes [10]. Typically, an ESD team becomes involved with discharge planning with patient, family and stroke unit staff at an early stage of admission. Prior to discharge from hospital, a member of the team may undertake a home visit (with the patient) or an environmental visit (without the patient). Rehabilitation and ongoing care provided by a specialist ESD team begins immediately after discharge. The duration and intensity of ESD therapy depends upon patient need. The discharge policy of ESD services varies, with some services defining a maximum period of input of three months. ESD teams do not usually retain any contact or involvement with patients once their input has ceased. Following discharge from ESD services, the concept of organised stroke care disappears. Patients who have ongoing rehabilitation needs may be referred to a range of services, most of which do not offer specialist stroke rehabilitation, such as neurorehabilitation teams, day hospitals and community rehabilitation services. This research study is evaluating an extended stroke rehabilitation service which commences when ESD ends. The service is delivered by existing ESD teams and extends organised stroke care beyond ESD.

## Methods/design

### Study aim and objectives

This study aims to determine the clinical and cost effectiveness of an extended stroke rehabilitation service. The objectives of the study are as follows:

1. To determine whether an extended stroke rehabilitation service (intervention) improves patient outcomes compared to usual care (control). The primary outcome is extended activities of daily living (EADL) at 24 months following randomisation. Secondary outcomes are: health status, quality of life, mood and experience of services (12 and 24 months following randomisation).
2. To determine whether an extended stroke rehabilitation service improves carer outcomes compared to usual care. Outcomes are: quality of life, carer stress and experience of services (12 and 24 months following randomisation).
3. To determine the cost effectiveness of an extended stroke rehabilitation service.
4. To document how the extended stroke rehabilitation service is implemented and delivered in different settings.
5. To seek the views and experiences of patients, carers and rehabilitation staff about the community rehabilitation they have received or provided.
6. To explore the impact of the severity of activity limitation, pre-stroke heath status and comorbidity upon the effectiveness of the intervention.

### Study design

**Figure 1:**
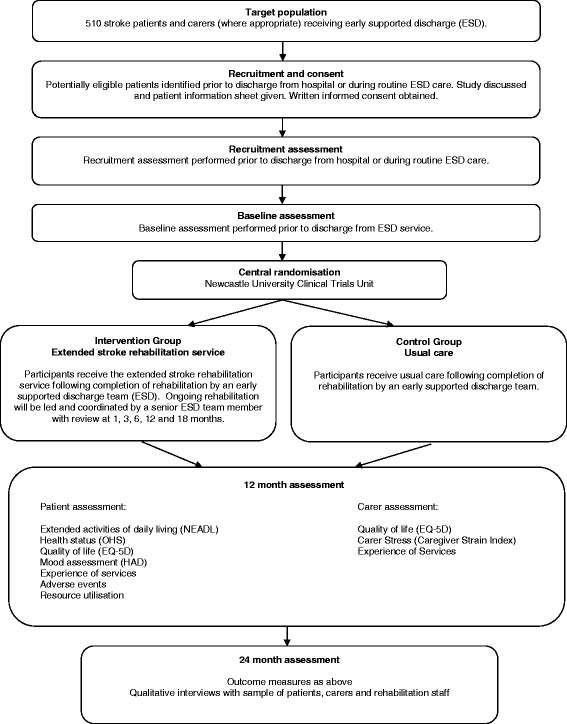
Study summary. ESD: Early Supported Discharge. NEADL: Nottingham Extended Activities of Daily Living Scale. OHS: Oxford Handicap Scale. HAD: Hospital Anxiety and Depression Scale.

This study is a pragmatic, observer-blind, parallel group, multicentre, randomised controlled trial with health economic and process evaluations. Figure 1 summaries the study methods.

### Study setting

The study is being conducted in National Health Service (NHS) stroke services that provide ESD. To be eligible to take part, ESD services must meet the following criteria:

1. The ESD service is a multidisciplinary stroke team who provide community rehabilitation following discharge from hospital.
2. The ESD service provides stroke rehabilitation at home within 48 hours of patient discharge from hospital.
3. The ESD service provides stroke rehabilitation for a specified period of time and/or has clear criteria for discharge of patients from the service.

### Study participants

Adults who have experienced a stroke and who fulfil the following criteria are eligible:

#### Inclusion criteria

1. Aged 18 years and over,
2. Confirmed diagnosis of new stroke (first ever or recurrent),
3. Will be discharged from hospital under the care of an ESD team or are currently receiving this service.

#### Exclusion criteria

1. Unable to participate in a rehabilitation programme which focuses upon EADL

A carer is the main family member or friend, who will provide support after discharge. He or she may not necessarily be co-resident with the patient. If a stroke patient has no carer or a carer does not wish to participate in the study, the patient may still participate in the study.

### Case ascertainment, recruitment and consent

#### Patients

Potential patients are identified and recruited by NHS staff (clinicians, staff from the Local Clinical Research Network (LCRN) and senior members of the ESD team) at each participating NHS centre. Potential patients may be recruited prior to discharge from hospital or whilst receiving care from an ESD service. Although the extended stroke rehabilitation service does not commence until routine ESD services end, identification and recruitment of patients in hospital or during ESD maximises recruitment opportunities.

#### Consent for patients with mental capacity

For potential participants with mental capacity to consent to research, NHS staff approach the patient, discuss the study and provide a study information sheet. After allowing sufficient time for potential participants to decide whether to take part in the study (over 24 hours) and an opportunity to ask questions, consent is obtained in writing. Where a patient has mental capacity but is unable to sign the consent form (for example, because of weakness of the dominant hand following stroke), consent is confirmed orally in the presence of a witness (an individual not otherwise involved in the trial), who signs the consent form on behalf of the participant.

#### Consent for patients with aphasia

In order to include stroke patients with communication difficulties due to aphasia in this study, a set of ‘easy access’ study documentation was specifically developed. To recruit and consent patients with aphasia, NHS staff approach the patient, discuss the study and provide them with the easy access information sheet. After allowing sufficient time for the information to be considered (over 24 hours) and an opportunity to ask questions, consent is obtained in writing using the easy access consent form.

#### Consent for patients without mental capacity

Stroke patients who do not have mental capacity can also take part in this study. As patients with mental incapacity often have more disabling strokes, we believe that they may benefit from the extended stroke rehabilitation service. To recruit patients lacking in mental capacity, NHS staff identify a personal consultee to approach and discuss the study with. This is a person who is in a position to advise on the wishes and feelings of the potential patient in relation to taking part in this research project. The identified consultee is provided with a consultee information sheet. After allowing sufficient time to consider the patient’s wishes and feelings (over 24 hours) and an opportunity to ask questions, the consultee is asked to complete a consultee declaration form if they believe the patient would have no objection to taking part in the study.

If a patient regains capacity during their participation in the study, they will be informed about the study, given a ‘recovered capacity’ patient information sheet and asked to provide their own consent to continue in the study on a recovered capacity consent form. If a patient does not wish to continue in the study, they will be withdrawn. Data collected prior to withdrawal will be used in the study analysis.

Due to the nature of this study, potential patients lacking in capacity also need to have a relative or friend (carer) who is prepared to assist with the extended stroke rehabilitation service reviews and outcome assessments, as these are unlikely to be possible without their support.

#### Loss of capacity to consent to research during participation in the study

If a patient who has provided their own consent loses capacity to consent to research during their participation in the study, advice will be sought from a personal consultee about their continuing participation in the study. This will be a person who is in a position to advise on the wishes and feelings of the patient in relation to the research study. On entering the study, patients are asked to nominate a relative or friend who they would like to be their consultee should they lose capacity to consent to research. In the event of loss of capacity to consent to this research, the nominated consultee will be given a ‘loss of capacity’ consultee information sheet. If a consultee believes the patient would have no objection to continuing in the study, they will be asked to complete a loss of capacity consultee declaration form. If the consultee believes that the patient would not wish to continue in the study, the patient will be withdrawn from the study. Data collected prior to withdrawal will be used in the study analysis.

#### Carers

Potential carers are identified by ESD senior team members whilst the patient is receiving routine ESD care. At the time of patient discharge from routine ESD services, if the patient has an identified carer, he or she is provided with an invitation letter, study information sheet, study carer baseline questionnaire and pre-paid envelope (addressed to the study coordinating centre). Provision of the invitation letter and study documents may be in person by an ESD senior team member, by post by the local study team or by a consented patient. These three options are used to maximise potential opportunities for carers to take part in the study as carers are not always present at staff visits. The invitation letter asks a carer to complete and return the baseline questionnaire, if he or she is willing to participate in the study.

### Recruitment assessment

A patient recruitment assessment is performed by NHS staff after informed consent has been obtained and within four days prior to planned discharge from hospital, or during routine ESD care. The following data are collected: demographic data, pre-stroke level of EADL (Nottingham EADL Scale [11]), pre-stroke health status (Oxford Handicap Scale [12]), date of hospital admission, date of stroke, stroke type and subtype, National Institute of Health Stroke Scale (NIHSS) [13], comorbidity and pre-stroke resource usage (adaptation of the Client Service Receipt Inventory (CSRI) [14-16]).

### Baseline assessment

A patient baseline assessment is performed by NHS staff at discharge from routine ESD services and immediately prior to randomisation. The following data is collected: date of hospital discharge, date of ESD discharge, Abbreviated Mental Test Score [17], Sheffield Aphasia Screening Test [18], EADL (Nottingham EADL Scale [11]), health status (Oxford Handicap Scale [12]), quality of life (EuroQol EQ-5D [19]) and mood (Hospital Anxiety and Depression Scale [20]).

Carers receive a baseline questionnaire with the study invitation letter. The questionnaire collects the following data: demographic data, quality of life (EuroQol EQ-5D [19]) and carer stress (Caregiver Strain Index [21]).

### Randomisation

Randomisation is by a central independent web-based service hosted by Newcastle University Clinical Trials Unit. Participants are stratified according to stroke service and randomised to either the intervention or control group in a 1:1 ratio using permuted block sequences. Stroke patients and carers are randomised as a single unit.

### Study control treatment

#### Usual care and provision of booklet about stroke care and rehabilitation

Stroke patients in the control group receive usual ESD care with subsequent referral to other rehabilitation services post-discharge from ESD if required and in accordance with usual care. Patients who have ongoing rehabilitation needs following completion of ESD may be referred to a range of services such as neurorehabilitation teams, day hospitals and community rehabilitation services.

In addition, both control and intervention group participants receive the booklet ‘Care after stroke or transient ischaemic attack. Information for patients and their carers’, written by the Intercollegiate Stroke Working Party [22]. It describes what a stroke is, assessment, acute management and rehabilitation. It is based on the National Clinical Guideline for Stroke [6].

### Study intervention treatment

#### Extended stroke rehabilitation service and provision of booklet about stroke care and rehabilitation

Stroke patients in the intervention group receive an extended stroke rehabilitation service for 18 months following completion of rehabilitation with their ESD team. This is in addition to usual care. They also receive the Intercollegiate Stroke Working Party booklet about stroke care and rehabilitation.

The extended stroke rehabilitation service consists of reviews by a designated senior member of the ESD team at 1, 3, 6, 12 and 18 months post-discharge from routine ESD. We have chosen to evaluate a model where care is coordinated rather than delivered by a senior member of the ESD team, as this model could potentially be delivered by the National Health Service (NHS) throughout the UK, if shown to be effective. The role of specialists coordinating rather than delivering rehabilitation has been shown to be effective in other conditions [23].

Each review consists of:

1. A semi-structured interview to identify the patient’s progress, current rehabilitation needs and service provision. The interview addresses everyday activities (personal care, meal times, domestic activities, indoor mobility, outdoor mobility, shopping, hobbies and driving), social participation and wider issues (mood, memory, pain, communication and medical issues) which may be problematic for stroke survivors. The views of both the patient and carer (where appropriate) are sought.
2. Joint rehabilitation goal setting. From the identified progress and rehabilitation needs, up to five individual rehabilitation goals are set by the patient (and carer) in collaboration with the senior ESD team member who conducts the review. The focus of joint goal setting is upon increasing participation in everyday activities. The physical, psychological and social factors which may impact on goal attainment are considered. At each review, progress towards goals from the previous review is assessed prior to further goal setting. Achievement of goals is recorded using a Goal Attainment Scale [24].
3. Action planning. The patient (and carer) agree an action plan for each rehabilitation goal. This may include:
  Verbal advice and encouragement;
  Discussion with the stroke team, rehabilitation team, primary care team or social services involved in care;
  Signposting to local activities, community organisations or voluntary services;
  Referral to stroke services, rehabilitation services or primary care services for further assessment and treatment, if required, according to local guidelines and/or service provision.

The majority of the reviews are intended to be done by telephone. The senior ESD team member will know the patient and carer as he or she will have treated the patient as part of the ESD service. However, if the patient and/or carer is unable to participate in a telephone review, a home visit can be undertaken. Patients are given a study appointment card which also contains a short checklist of rehabilitation issues to be covered in each review. This is to allow patients (and carers) time to consider the topics to be discussed prior to the interview. Patients with aphasia receive an easy access version of the appointment card.

All senior ESD staff taking part in the trial receive an extended stroke rehabilitation service manual and training in delivery of the new service. The extended stroke rehabilitation manual describes how to conduct the reviews, including guidance on exploring rehabilitation needs, goal setting and appropriate interventions to meet a patient’s needs.

Subsequent to each review the ESD therapist or nurse may contact services currently involved in the patients care to discuss progress, goals and care plan.

A summary of the review and recommendations for rehabilitation is sent to the patient, patient’s GP, stroke physician and therapists currently involved in the patient’s care. Patients with aphasia receive an easy access version of the letter.

### Outcome assessments

Outcomes are assessed at 12 months (+/− 7 days) and 24 months (+/− 7 days) following randomisation.

Patient outcome assessments are undertaken by telephone by a researcher based in the study coordinating centre. For participants who do not have a telephone or who are unable to communicate by telephone, outcomes are collected by postal questionnaire. If a patient is unable to participate in a telephone interview or complete a postal questionnaire, outcome assessments are undertaken by local staff trained by the study team.

The following data is collected from patients: EADL (Nottingham EADL Scale [11]), health status (Oxford Handicap Scale [12]), quality of life (Euroqol EQ-5D [19]), mood (Hospital Anxiety and Depression Scale [20]), experience of services (adaption of an experience survey designed by Northumbria Healthcare NHS Foundation Trust) and resource utilisation (adaptation of the CSRI [14-16]).

Carers’ outcome assessments are undertaken by postal questionnaire. This is because the Caregiver Strain Index asks some sensitive questions about the impact of stroke upon the carer [21]; a carer may modify their answer if they could be overheard on the telephone by the patient. The carer outcome questionnaire collects the following data: quality of life (EuroQoL EQ-5D [19]), carer stress (Caregiver Strain Index [21]) and experience of services (adaption of an experience survey designed by Northumbria Healthcare NHS Foundation Trust).

### Blinding

Due to the nature of the intervention, it is not possible to blind stroke patients or carers to treatment allocation. Where patient outcome assessments are undertaken by telephone or face to face, it is intended that they are conducted blinded to treatment allocation. After each assessment the assessor is asked to record whether they have unintentionally become aware of treatment allocation due to conversation with the participant. Success of patient outcome assessment blinding will be reported.

### Study withdrawal

No specific study withdrawal criteria have been pre-set. Stroke patients and/or carers may withdraw from the study at any time for any reason. Data collected prior to withdrawal will be used in the study analysis unless consent for this is specifically withdrawn. Reasons for withdrawal are sought, but patients and/or carers can chose to withdraw without providing an explanation. Investigators, senior ESD team members and/or a patient’s consultee (in the case of mental incapacity) may also withdraw participants from the study at any time if they feel it is no longer in their interest to continue, for example, because of intercurrent illness or adverse events.

### Safety evaluation

The safety of the extended stroke rehabilitation service is being evaluated by examining the occurrence of all adverse events and serious adverse events in accordance with National Research Ethics Committee (NRES) guidance.

### Statistical analysis

#### Primary analysis

The primary outcome is the Nottingham EADL score [11] at 24 months. Analysis will be on the basis of intention to treat. Mean scores will be compared between intervention and control groups using multiple linear regression, including terms for sites and patient-level covariates such as baseline scores.

#### Secondary analyses

Secondary outcomes will be compared between intervention and control groups using multiple linear regression, including terms for sites and patient-level covariates such as baseline scores. Further exploratory descriptive analyses will examine the impact of the severity of activity limitation, pre-stroke health status and comorbidity upon the effectiveness of the intervention; there is not sufficient power to perform any formal subgroup analyses.

#### Sample size

There is consensus that a difference of 6 points (range: 0 to 66, standard deviation (SD): 18) on the Nottingham EADL Scale is clinically important, and power calculations for previous multicentre rehabilitation trials have been based upon this difference [16,25]. Responses from 382 patients split equally between intervention and control groups will provide 90% power to detect a difference in mean Nottingham EADL score of 6 points. Based on attrition in other stroke rehabilitation trials, we believe that there may be up to 25% attrition between study randomisation and the 24-month (primary) outcome assessment. To allow for this, we aim to randomise 510 participants into the study.

Although participants may be recruited any time from within four days prior to discharge from hospital until discharge from ESD, many are likely to be recruited prior to discharge from hospital. There may be several weeks between recruitment and randomisation, and some participants may withdraw from the study during this time. Our current estimate is that up to 15% may drop out before randomisation. The target recruitment sample size is being kept under review, and recruitment will cease when we estimate that 510 participants will be randomised. Reasons for loss from the trial are recorded.

### Economic analysis

The economic evaluation will include both a cost-effectiveness analysis (CEA) and a cost-utility analysis (CUA) [26]. The CEA will be undertaken using the Nottingham EADL Scale [11] at 24 months as the measure of effect. The result of the CEA will be an incremental cost-effectiveness ratio (ICER) [27]. In order to quantify the uncertainty associated with the ICER, the stochastic analysis will be conducted, with the results presented as a cost-effectiveness acceptability curve (CEAC) [28]. The CEAC will show the probability that an extended stroke rehabilitation service is cost effective compared with usual post-ESD care, given the observed data, for a range of maximum monetary values that decision-makers may be prepared to pay for unit change in Nottingham EADL Scale [11]. The CUA will compare changes in health-related quality of life, based on responses to the EuroQoL EQ-5D [19], at baseline and 24 months across both arms of the trial. These data will be combined with study participant’s mortality to estimate quality-adjusted life years (QALYs). Both costs and QALY data will be combined into an incremental cost per QALY. Both analyses will be carried out from the perspective of the NHS, but we will also take societal perspective by including costs borne by the participants themselves and their informal carers by obtaining information about time away from employment and time spent providing care.

Resource utilisation will be assessed at 12 and 24 months using an appropriate adaptation of the CSRI [14-16]. We will identify all the relevant financial costs associated with providing the intervention. Relevant costs will be categorised as either fixed costs or variable costs, where fixed costs are those resources that are required to set up and run the service and variable costs are those required to treat an individual patient. Where appropriate, discounting [26] will be applied to financial costs and outcomes. Financial costs will be attached to the items of service used using data from the Personal Social Services Research Unit [29]. Because the consequences of an extended stroke rehabilitation service may extend beyond the 24-month timeframe of the trial, and may have on-going significant financial cost and quality of life implications (for example, a reduced incidence of hospitalisation related to falls or need for residential or nursing home care), a Markov model will be developed that can be used to predict outcomes up to 60 months based on the status at 24 months. Transition probabilities and cost associated with each state in the model will be obtained from published literature and, where no published evidence exists, expert opinion will be sought. Other forms of uncertainty such as variation in unit prices will be explored within the deterministic sensitivity analyses; where appropriate these CEACs will also be produced for these analyses.

### Parallel process evaluation

Parallel process evaluations of complex interventions being tested by randomised controlled trials are increasingly recommended [30]. They can provide information about unanticipated consequences, reasons for success, how an intervention can be improved and identify contextual factors associated with variations in outcome [31]. This process evaluation is investigating the operation of the extended stroke rehabilitation service collecting both quantitative and qualitative data. This includes:

1. Mapping the rehabilitation and follow-up services provided for stroke patients in each site.
2. Documenting how the new service is implemented and delivered in different settings. A senior ESD team member at each site completes a standard pro forma at each assessment. This consists of a progress update, rehabilitation undertaken since the previous assessment, services received and progress toward rehabilitation goals. The patient’s current rehabilitation needs, assessment and advice given to the patient and carer are recorded. Details of new referrals to other services are also recorded.
3. Seeking the views and describing the experiences of patients and carers about the rehabilitation services they received. Semi-structured interviews will be conducted with a purposive sample of patients and caregivers who received the extended stroke rehabilitation service and usual care. Sampling will use variables of age, gender, level of disability and geographical location of research sites for participant selection. Equal numbers of control and intervention group participants will be selected to allow comparison between the two groups. A topic guide has been devised drawing on relevant literature [32-35]. The interviews include questions on views and perceptions about ability to undertake EADL, enablers and barriers, perceptions of provision of rehabilitation to support EADL and perceptions of unmet need. Interviews will take place after final outcome assessment. The topic guide will be refined by pilot interviews with a small sample (approximately four) of patients and their carers. The guide will then be used with up to 60 patients and/or patient/carer dyads, with final numbers determined when data saturation is considered to have been achieved through ongoing analysis [36]. All interviews will be digitally recorded, transcribed and entered onto NVivo (QSR International, Melbourne, Australia) for data management.
4. Seeking the views and experiences of senior members of the ESD teams and community rehabilitation staff about the services provided to the intervention and control groups. Semi-structured interviews will be conducted with a purposive sample of rehabilitation staff who were involved in the study. Sampling will ensure the involvement of the range of healthcare professionals delivering the intervention. A topic guide will be developed following a small pilot sample of unstructured interviews with two senior ESD team members who provided the extended stroke rehabilitation service and two members of community rehabilitation services. Interviews will investigate their experience in delivering rehabilitation to improve EADL, the longer term needs relating to the EADL of people with stroke and their carers, and views about the extended stroke rehabilitation service compared to usual care. Interviews will then be undertaken with two to three members of the community teams in all study sites. Since interviews conducted during the trial may have the undesired effect of changing staff practice, these will be undertaken within six weeks of the end of delivery of the trial intervention.

### Interview data analysis

Transcribed interviews will be checked and corrected for errors by the interviewer. Analysis will follow standard approaches that entail familiarisation with the material, coding and category development to identify the main patterns of responses, consistencies and divergences across and within interviews, and to identify similarities and differences between and within group testing [37]. Common experiences, outlier views and significant differences by category of respondent will be identified. A sub-sample of interview data will be independently analysed by a study co-investigator, and compared to the analysis undertaken by the interviewer.

We will use accepted approaches to demonstrating rigour in qualitative research [38], including clear documentation of research methods and processes, transparency in the use of data collection schedules, independent coding and analysis by researchers, systematic exploration of alternative explanations for the processes claimed to explain our findings and, as far as possible, the involvement of study participants in a discussion of the initial analyses.

### Ethics and regulatory issues

The study sponsor is Northumbria Healthcare NHS Foundation Trust. The study is being conducted in accordance with Research Governance Framework for Health and Social Care [39]. Ethical approval was granted by the National Research Ethics Committee North East - Newcastle and North Tyneside 1 (reference 12/NE/0217). NHS Trust approvals have also been granted from Northumbria Healthcare NHS Foundation Trust, Leeds Teaching Hospitals NHS Trust, Leeds Community Healthcare NHS Trust, Newcastle upon Tyne Hospitals NHS Foundation Trust, Pennine Acute Hospitals NHS Trust, Pennine Care NHS Foundation Trust, South Tyneside NHS Foundation Trust, Royal Cornwall Hospitals NHS Trust, Solent NHS Trust, Portsmouth Hospitals NHS Trust, Plymouth Community Healthcare, Norfolk Community Health and Care NHS Trust, Staffordshire and Stoke on Trent Partnership NHS Trust, Royal Bournemouth and Christchurch Hospitals NHS Foundation Trust, Hull and East Yorkshire Hospitals NHS Trust, Humber NHS Foundation Trust, York Teaching Hospital NHS Foundation Trust, Sherwood Forest Hospitals NHS Foundation Trust, Somerset Partnership NHS Trust, Wrightington, Wigan and Leigh NHS Foundation Trust and Cardiff and Vale University Health Board.

## Discussion

The provision of longer term support for stroke survivors and their families is currently limited. Well-designed randomised controlled trials are required to provide an evidence base to inform development of community stroke services. The EXTRAS trial is a large, multicentre, randomised controlled trial to evaluate an extended stroke rehabilitation service which begins once ESD is completed. The results from the trial will inform future stroke service planning and configuration.

## Trial status

The EXTRAS trial commenced recruitment in November 2012. It is currently running in 18 NHS study centres. One or more centres are open in North East England, North West England, Yorkshire, Midlands, East England, South East England, South West England and Wales. The EXTRAS trial has recruited 541 patients at the time of submission of this manuscript (February 2015). Recruitment is scheduled for completion in summer 2015. Follow-up will continue until 2017. Results will be submitted for publication in 2018.

## Abbreviations

ADL: Activities of Daily Living
CEA: Cost-effectiveness analysis
CEAC: Cost effectiveness acceptability curve
CSRI: Client Service Receipt Inventory
CUA: Cost-utility analysis
EADL: Extended Activities of Daily Living
ESD: Early Supported Discharge
ICER: Incremental cost effectiveness ratio
LCRN: Local Clinical Research Network
NIHSS: National Institute of Health Stroke Scale
NHS: National Health Service
NRES: National Research Ethics Service
QALY: Quality Adjusted Life Years
UK: United Kingdom
